# A novel introgression line collection to unravel the genetics of climacteric ripening and fruit quality in melon

**DOI:** 10.1038/s41598-021-90783-6

**Published:** 2021-05-31

**Authors:** Lara Pereira, Miguel Santo Domingo, Jason Argyris, Carlos Mayobre, Laura Valverde, Ana Montserrat Martín-Hernández, Marta Pujol, Jordi Garcia-Mas

**Affiliations:** 1 Centre for Research in Agricultural Genomics (CRAG) CSIC-IRTA-UAB-UB, Edifici CRAG, Campus UAB, 08193 Bellaterra, Barcelona Spain; 2 Institut de Recerca i Tecnologia Agroalimentàries (IRTA), Edifici CRAG, Campus UAB, 08193 Bellaterra, Barcelona Spain

**Keywords:** Natural variation in plants, Plant breeding, Plant genetics

## Abstract

Introgression lines are valuable germplasm for scientists and breeders, since they ease genetic studies such as QTL interactions and positional cloning as well as the introduction of favorable alleles into elite varieties. We developed a novel introgression line collection in melon using two commercial European varieties with different ripening behavior, the climacteric *cantalupensis* ‘Védrantais’ as recurrent parent and the non-climacteric *inodorus* ‘Piel de Sapo’ as donor parent. The collection contains 34 introgression lines, covering 99% of the donor genome. The mean introgression size is 18.16 Mb and ~ 3 lines were obtained per chromosome, on average. The high segregation of these lines for multiple fruit quality traits allowed us to identify 27 QTLs that modified sugar content, altered fruit morphology or were involved in climacteric ripening. In addition, we confirmed the genomic location of five major genes previously described, which control mainly fruit appearance, such as mottled rind and external color. Most of the QTLs had been reported before in other populations sharing parental lines, while three QTLs (*EAROQP11.3*, *ECDQP11.2* and *FIRQP4.1*) were newly detected in our work. These introgression lines would be useful to perform additional genetic studies, as fine mapping and gene pyramiding, especially for important complex traits such as fruit weight and climacteric ripening.

## Introduction

Understanding the genetics beyond interesting traits, from human diseases to crop yield, has been one of the main goals of modern science. Genetic variation is studied and correlated with phenotypes, in order to identify the genomic regions controlling traits of interest. In plants, due to the ease of crossing, many types of segregating populations can be developed depending on the goal of the research and the availability of time and resources. In species with short generation periods, as cereals and vegetable crops, populations with increased complexity are usually developed, as Recombinant Inbred Line (RIL)^1^, Nested Association Mapping^2^, Multi-parent Advanced Generation Intercrosses^3^ and Introgression Line (IL)^4,5^ populations. IL collections are immortal lines that share a high proportion of genetic background from a recurrent parent, differing only in a specific region of the genome (introgression), inherited from the donor parent. Ideally, the complete genome of the donor parent is covered by the ILs conforming the collection. They are considered a powerful resource since, besides Quantitative Trait Loci (QTL) mapping experiments, they allow to perform subsequent specific studies as QTL validation^6^, QTL interactions^7,8^ and fine mapping^9,10^.

IL populations (also referred as Near Isogenic Lines, NILs) have been used as genetic resources in multiple species for several decades^11–13^, from model plants as *Arabidopsis thaliana*^14^, rice^15^ and tomato^16^ to less studied and even orphan crops, as strawberry^17^, peach^18^ and groundnut^19^. They have proven their efficiency to map and characterize traits related with disease resistance, plant architecture and fruit morphology and quality. Furthermore, they have been commonly used in breeding programs to introduce desired exotic alleles from donor accessions, where the recurrent parent is typically an elite line.

Generally, ILs are developed by genotyping the target introgression as well as the recurrent parent background with molecular markers. The rapid increase in marker availability and high-throughput genotyping techniques have changed the methods used to develop these populations. Recently, the trend has been to minimize the number of needed backcrosses by increasing the size of the initial screened populations and the number of markers, in order to decrease the number of non-desired contaminations in narrow non-genotyped regions of the genome^5,17,18^.

IL populations have been developed and widely used throughout the last years in melon^4,9,10,20–26^. Melon is a diploid species with a relatively small genome (450 Mb) and 2n = 2x = 24^27^. Melon has become an alternative model species to study certain traits, as climacteric ripening^28^ and sex determination^29^. An IL collection, developed from a cultivated variety (spp. *melo*) as recurrent parent and an exotic Korean accession (spp. *agrestis*) as donor parent^4^, was used to fine map and clone two genes controlling climacteric ripening^9^ and resistance to *Cucumber Mosaic Virus*^10^. In addition, pyramiding of multiple QTLs controlling or modulating the same trait was achieved through crosses of several ILs; e.g., the QTL *ETHQV6.3*, corresponding to *CmNAC-NOR*^9^ and involved in climacteric ripening, interacts with a second QTL (*ETHQB3.5*), leading to a more intense climacteric phenotype when both introgressions are combined in the same IL^23^.

Most of the populations developed in melon have been funded by a cross between an elite cultivar and an exotic accession. A recent study using a RIL population between two European cultivars, “Védrantais” (Ved), a typical French variety belonging to the *cantalupensis* group, and “Piel de Sapo” (PS), a Spanish variety from the *inodorus* group, showed segregation for traits related with fruit quality and climacteric ripening^30–32^. Climacteric ripening is a complex and polygenic trait that partially determines the overall fruit quality. Melon is one of the few species where both climacteric and non-climacteric varieties coexist, offering an excellent model to dissect the genetics of the trait^28^. Climacteric ripening is defined by an autocatalytic synthesis of the plant hormone ethylene at the onset of the ripening stage, followed by a peak of respiration^33^. In climacteric melons, these biochemical signals trigger multiple phenotypic changes such as chlorophyll degradation, production of several volatiles that lead to a sweet aroma, abscission layer formation in the pedicel and degradation of cell wall resulting in a loss of flesh firmness^34^.

The aim of this work was to develop an IL collection using Ved as recurrent parent and PS as donor parent in order to identify and validate QTLs controlling fruit quality and climacteric ripening in melon. The novelty of this IL collection is that both the recurrent (Ved) and the donor (PS) are elite cultivars that segregate for many traits. Ved is a medium-size rounded melon, with rind sutures, orange flesh, highly aromatic and climacteric ripening, while PS has bigger size, with elongated shape, mottled rind, white flesh, and non-climacteric ripening. This advanced germplasm resource would be useful in the future to further characterize these genetic factors through fine mapping and QTL interaction studies.

## Results and discussion

### Parent phenotypes

Two commercial lines, Ved and PS, were selected as parents to develop the IL collection due to the segregation of several relevant traits among them, such as ripening behavior and external appearance. They represent the two most common and consumed melons in Europe. Ved produces a round, medium-size fruit, whereas PS fruits are elongated and its fruit weight is higher (Supplementary Fig. S1A). The soluble solid content and fruit firmness are slightly higher in PS although not significantly different from Ved. The external appearance of the fruit also varies: Ved presents white/cream rind with marked sutures and PS mottled green rind (Supplementary Fig. S1B). The flesh color is orange in Ved and white in PS.

Interestingly, these two varieties represent the two extremes of ripening behavior in melon^31^. Ved is a typical climacteric variety, presenting the characteristic ethylene peak at the onset of ripening closely followed by classical phenotypic consequences such as intense sweet aroma, abscission layer formation, chlorophyll degradation and loss of flesh firmness. PS is completely non-climacteric, the ethylene production during ripening remains undetectable and it lacks the phenotypic changes associated to climacteric ripening, except loss of flesh firmness, a trait partially ethylene-independent^34^.

### Development of the PS IL collection

IL collections are a highly valuable resource for both research and breeding, although their development is more complex and expensive than other populations. In the last years, many IL collections have been described for different species, including melon, which have been successfully used to map and characterize QTLs^35–37^. Here, we developed a new IL collection to gain insight about the genetic control of fruit quality.

**Figure 1:**
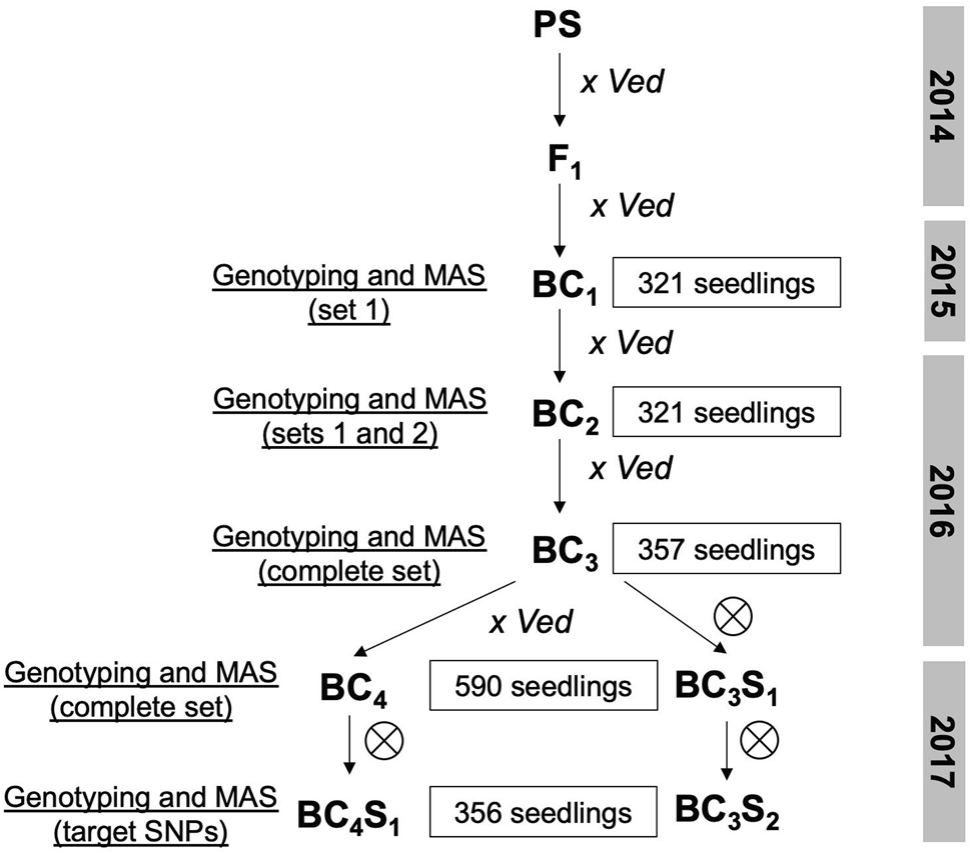
Breeding scheme followed to develop the IL collection.

In the BC_1_ population, the number of introgressions ranged from 4 to 17 per line, with a mean of 11.5 and a median of 12, indicating that on average one recombination per chromosome and meiosis had occurred. The use of marker-assisted selection in the first BC generation reduced substantially the risk of carrying non-detected contaminations. The percentage of Ved genome ranged from 13.0 to 80.1%, with an average of 48.7%. The observed heterozygosity was H_0_ = 0.46 and 16 SNPs showed significant segregation distortion. The highest segregation distortion was observed in the centromeric region of chromosome 12 (χ^2^ = 43.3), which presented a minimum observed heterozygosity of 0.31. A set of 25 BC_1_ plants (7.8%) were selected, presenting a mean of 8 introgressions and 62.2% of Ved background genome. After two more cycles of backcross and marker assisted selection to BC_2_ and BC_3_ (Fig. 1), the number of introgressions in the 64 selected lines was notably reduced to a mean of 2.6 introgressions and they carried an 85.7% of Ved genome on average. In the next generation, a set of 100 plants were selected, carrying 1.8 introgressions and 88.3% of Ved genome on average, and most of the final ILs were recovered from their progenies. Due to various complications (misbehaved SNPs, loss of plants due to pests or bad fruit set), a 6% of PS genome remained uncovered in the IL collection. Lines from previous generations were used to obtain additional ILs, and three and two of them were phenotyped in 2019 and 2020, respectively.

**Figure 2:**
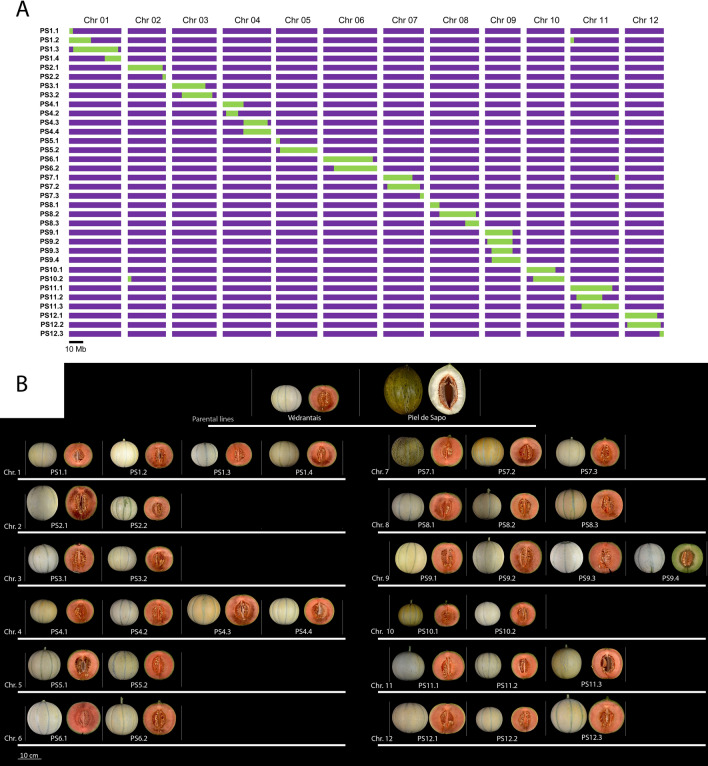
The IL collection. (**A**) Genotypic characterization of the IL collection. Purple represents the genotype of the recurrent parent Ved and green, the genotype of the donor parent PS. (**B**) Representative images of the ILs.

The most remarkable parameters for an IL collection are the number of ILs per chromosome, which is directly correlated with the resolution and with the required resources for phenotyping, the size of the introgressions, which also defines the resolution, and the number of generations needed to obtain the ILs, which is related with undesired introgressions in non-target regions. The PS IL collection, comprised by 34 lines, covered the complete genome of PS except the last 3.8 Mb of chromosome 3 (Fig. 2A and Supplementary Table S1). A mean of 2.8 ILs per chromosome was obtained, with a large range of introgression length from 2.46 to 35.32 Mb (Supplementary Table S2). Considering the overlapping of introgressions from different ILs, we could define 42 genomic bins. In comparison to other IL collections, this is an intermediate size, below the 6 ILs per chromosome obtained in a strawberry IL collection^17^ but higher than the 1.3 ILs per chromosome from other recently described melon IL collections^20,21^ (Supplementary Table S3). The size in genetic distance units, inferred from a genetic map in a RIL population using the same parents^30^, ranged from 14.7 to 129.2 cM, with an average of 62.8 cM. As we could expect, the introgressions in the telomeric regions were smaller in terms of physical distance but larger in genetic distance, as opposed to the introgressions covering the pericentromeric regions (Fig. 2A). The size of the introgressions was slightly higher than the genetic distance value obtained in other IL collections (mean of 38.7 cM), but similar in physical distance^4,5,17,20^. This discordance could be attributed to the strategy used to select the SNPs for the marker-assisted selection, which was mainly based on physical distances (markers homogeneously distributed across the chromosomes). The number of generations used to obtain the ILs in our work was generally six (BC_3_S_2_ and BC_4_S_1_), as in most other IL collections (Supplementary Table S3). The percentage of covered PS genome is ~ 99%, slightly higher than in other IL collections.

### QTL mapping

#### Fruit quality

Several traits contributing to fruit quality were segregating in the IL collection, including several external characteristics of the fruit as color and morphology (Fig. 2B, Supplementary Table S4). Qualitative phenotypes determining the external appearance of the fruit were already appreciated during the development of the IL collection. The mapping of these traits to the corresponding genomic region was easily performed even in pre-ILs due to its simple inheritance and high heritability, and the phenotyping of the complete collection performed in 2018, 2019 and 2020 confirmed the preliminary results.

**Figure 3:**
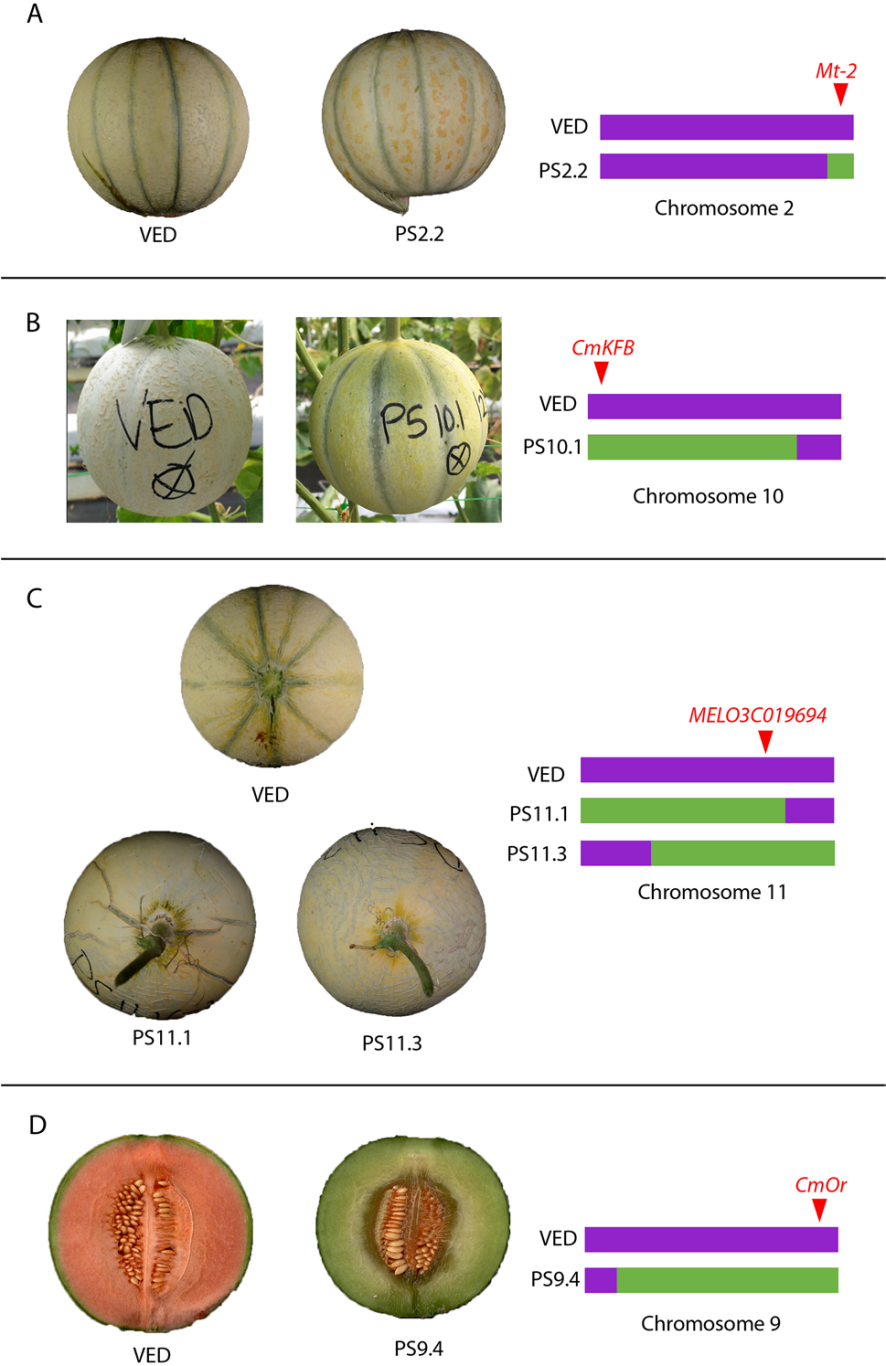
Segregation of qualitative traits and genomic location of the responsible major gene (**A**) Mottled rind (**B**) Yellowing of the rind (**C**) Presence of sutures (**D**) Flesh color of mature fruits.

PS2.2 presented mottled rind, observed as dark green spots during early stages of fruit development and turning to bright yellow spots in ripe fruits (Fig. 3A). This trait is mainly controlled by the major gene *Mt-2*, delimited to the interval 24.77–27.06 Mb on chromosome 2. *Mt-2* was also mapped to chromosome 2 in previous studies^30,38^.

PS7.1 and PS7.2 showed a dark green external color during fruit development as PS, contrasting to the rest of the IL collection which shared the white external color present in Ved. Similarly, this trait is mainly determined by one unique gene, previously described and designated *Wi*^39^, delimited by the overlapping region of both ILs, which is located within 2.62–20.72 bp. This interval colocalized with the region identified in the Ved × PS RIL population^30^.

PS10.1 fruits were white during fruit development as Ved but turned to a light-yellow during fruit ripening instead of cream (Fig. 3B). The interval of the gene primarily determining this trait (0–5.10 Mb on chromosome 10), contains *CmKFB*, which controls flavonoids biosynthesis in melon rind^40^.

PS11.1 and PS11.3 presented smooth rind, without sutures (Fig. 3C), in contrast to Ved and the rest of the IL collection. The responsible gene could be mapped to the interval shared by both lines on chromosome 11, from 21.26 to 29.79 Mb. *MELO3C019694*, located within the introgressions of PS11.1 and PS11.3, was proposed as responsible for the presence of sutures, due to a delay in gene expression during the initial stages of fruit development^41^. The same trait has been studied in other cucurbits, especially in *Cucurbita pepo*^42^, but the biological process causing the sutures has not been described yet.

**Figure 4:**
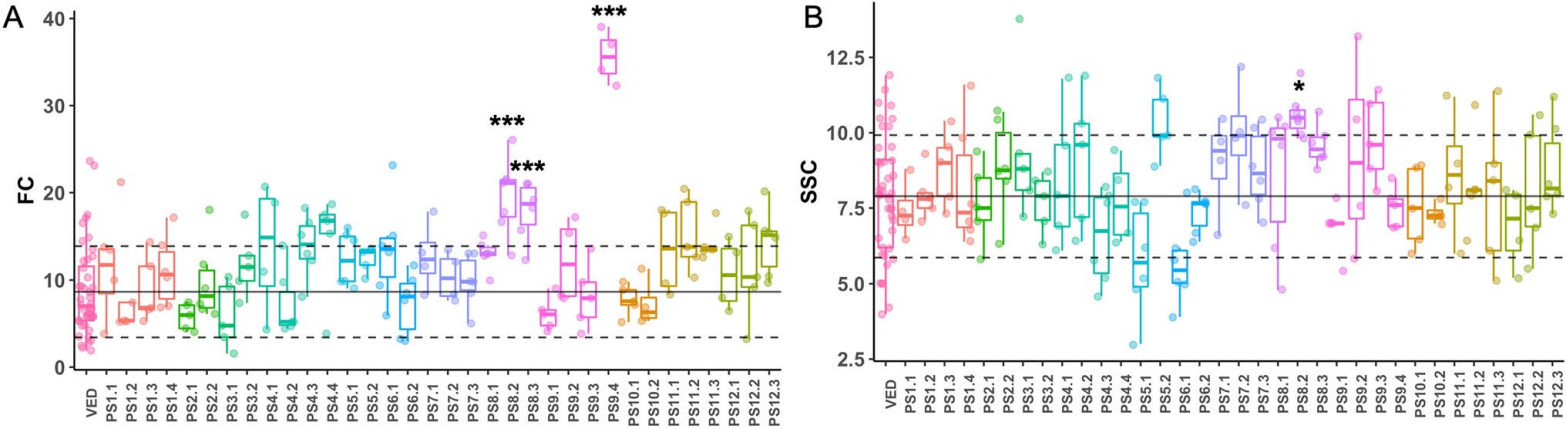
Boxplot representing FC and SSC values for ILs. Each dot represents a replicate, the black solid line corresponds to the average and the grey dashed lines to the average ± SD of the recurrent parent Ved. Significant differences are marked with asterisks: *< 0.05, **< 0.01, ***< 0.001.

PS9.4 was the only IL in our collection without orange flesh (Fig. 3D); however, unlike the donor parent PS, PS9.4 flesh is green. This phenotype is explained by the oligogenic inheritance of flesh color in ripe fruits. Three phenotypes, orange, white and green flesh, can be observed depending on the allelic combination of two genes that act epistatically. A dominant gene on chromosome 9 already cloned, *Gf* or *CmOr*^43^, controls the presence of orange color, conferred by a high content of carotenoids and is located in the position 21.68 Mb of chromosome 9 (version v3.6.1 of the melon genome). The non-orange allele, which is recessive, allows the manifestation of the second gene, called *Wf*, to control white (dominant) or green (recessive) flesh color^44–46^. *Wf* has been mapped using several populations to an interval of 5 Mb on chromosome 8^45,46^ and two genes, *MELO3C003069* and *MELO3C003097* have been proposed as possible candidate genes^41,44^. Ved genotype is *Gf/Gf wf/wf* and PS genotype is *gf/gf Wf/Wf*; therefore, the genotype of IL PS9.4, carrying a PS introgression in *Gf* locus, is the double recessive *gf/gf wf/wf*, leading to green color instead of the white color observed in PS. In addition to the visual inspection of flesh color, we also quantified CIELAB color parameters in Tomato Analyzer 4.0^47^. We then used the FC index (see “Materials and methods”), representing color differences between the ILs and Ved, with the goal of identifying additional minor QTLs. However, only the ILs carrying the two genes mentioned above were significantly different from Ved (Fig. 4A).

PS8.2 was the only IL in which soluble solid content significantly differed from Ved, with 10.59° Brix on average, representing an increase of ~ 34% compared to Ved (Fig. 4B, Table 1). This QTL was already described in the Ved × PS RIL population^30^. PS5.1, carrying an introgression from the beginning to 2.83 Mb of chromosome 5, reduced soluble solid content 34.3% when compared to Ved, dropping from 8.78° to 5.77° Brix, although it was not significant. This region in chromosome 5 colocalizes with a previously described QTL on chromosome 5, *sscqsc5.1*^37^; however, the PS allele, conferring lower soluble solid content in our IL collection, had the opposite effect in PS × “Trigonus” and PS × “Songwhan Charmi”^37^.

**Table 1:**
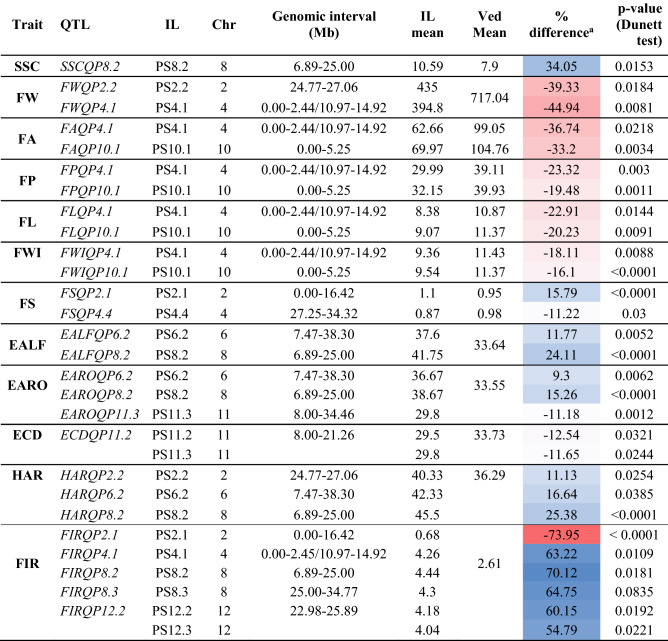
QTLs identified for quantitative traits in the IL population.

^a^The color gradient corresponds to the percentage of reduction (red) or increase (blue) in the IL phenotype when compared to Ved.

#### Fruit morphology

**Figure 5:**
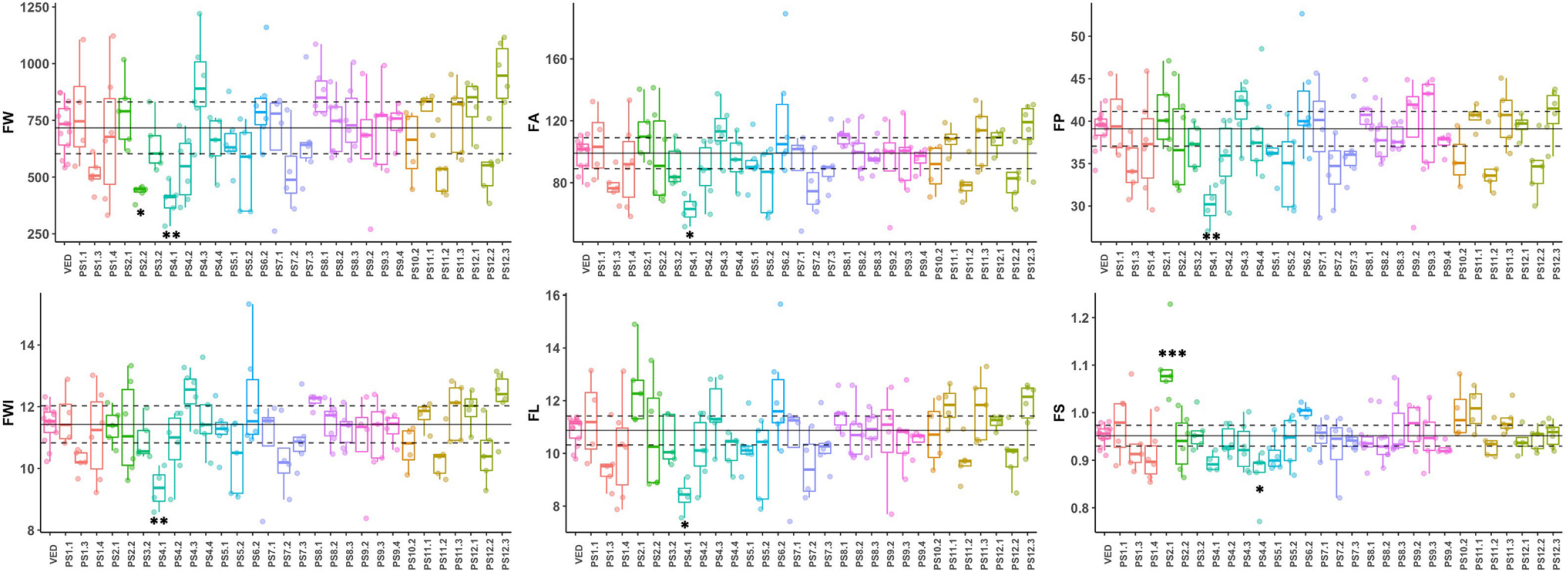
Boxplot representing the values of morphological traits for ILs in 2018. Each dot represents a replicate, the black solid line corresponds to the average and the grey dashed lines to the average ± SD of the recurrent parent Ved. Significant differences are marked with asterisks: *< 0.05, **< 0.01, ***< 0.001.

Fruit weight is one of the most relevant traits for the industry since it defines yield and also drives consumer’s preferences. Although a few genes controlling fruit weight have been cloned in the model fruit crop tomato^48–50^, the genetic basis of the trait in melon remains poorly understood. Several QTLs have been described in different populations through both QTL mapping and genome-wide association studies, however the underlying genes responsible of the phenotype remain unknown^51^. In the PS IL population, we identified two QTLs affecting fruit weight, carried by ILs PS2.2 and PS4.1 (Fig. 5). PS4.1 was also significantly different to Ved in other size-related traits as fruit area, fruit perimeter, fruit length and fruit width. In addition, PS10.1, evaluated in 2019, presented lower size overall and was significantly different from Ved for all size-related traits except fruit weight (Table 1 and Supplementary Fig. S2).

PS2.2 borne fruits with an average weight of 435 g, 39.3% reduction as compared to Ved (Table 1, Fig. 5 and Supplementary Fig. S3). *FWQP2.2* was delimited to the distal region of chromosome 2, from 24.77 to 27.06 Mb. No QTLs were identified for the other size-related traits in the same genomic region, although some of the fruits actually presented lower values (Fig. 5); these traits were more variable than fruit weight, possibly causing a lack of statistical power. A meta-QTL for fruit weight was defined previously on chromosome 2, colocalizing with the gene *a*, which causes sex determination in female flower. Thus, in those populations the fruit weight difference may be a consequence of sex determination^51,52^. Although the QTL described in our IL collection does not colocalize with *a* (*MELO3C015444*), indicating that the underlying gene has to be distinct, another QTL has been also reported at the distal part of chromosme 2 in a RNA-Seq-based QTL mapping^44^.

PS4.1 fruits weighed 394.8 g and showed a significantly smaller size than Ved (Table 1 and Fig. 5). The reduction in average values for the IL as compared to Ved was about 44.9% for fruit weight, 36.7% for fruit area, 23.3% for fruit perimeter, 18.1% for fruit width and 22.9% for fruit length. Since both dimensions were similarly decreased, the fruit shape was not affected. PS4.1 covers from the beginning of the chromosome 4 to 14.92 Mb; however, PS4.2, which did not show any fruit size difference compared to Ved, spans from 2.44 to 10.97 Mb. Therefore, *FWQP4.1* could be located either from 0 to 2.44 Mb or from 10.97 to 14.92 Mb (Supplementary Fig. S3). Although we do not know in which part of the introgression is the QTL, another QTL for fruit weight was previously reported in an IL population using PS as recurrent parent^24^, colocalizing with the second part of *FWQP4.1*, suggesting our QTL might be located in the 10.97–14.97 Mb interval.

PS10.1 carried fruits with a smaller size and a flattened shape in comparison to Ved. Fruit area, fruit perimeter, fruit length and fruit width were reduced between ~ 16 and 33%. Fruit weight was also lower than the rest of ILs but was not significant (Supplementary Fig. S2). These QTLs (*FAQP10.1, FPQP10.1, FLQP10.1* and *FWIQP10.1*) were located on chromosome 10 from 0 to 5.25 Mb (Table 1, Supplementary Fig. S3). It is not the first time that a QTL affecting fruit morphology is located on chromosome 10. In a similar study with ILs, but using different recurrent and donor parents, a QTL at the beginning of chromosome 10 was reported affecting fruit weight^24^.

Surprisingly, in these three ILs the alleles from PS were reducing fruit weight. Nevertheless, undoubtedly some alleles contributing to develop large fruits must be present in the PS genome, since its fruit weight is nearly double than Ved fruits. We observed a trend in four ILs which showed a median fruit weight higher than average plus one standard deviation of Ved fruit weight. PS4.3, PS8.1, PS12.1 and PS12.3 fruits weighed 905, 880, 812.82 and 916.67 g, respectively, representing increases in fruit weight between 16.3 and 31.1% as compared to Ved (Fig. 5). However, the values presented a substantial variation within each line, probably preventing from detecting significant differences. The larger size of these fruits was visually obvious (Fig. 2B) and also other size-related traits as fruit area and fruit perimeter showed this trend (Fig. 5).

Two QTLs were identified for fruit shape. PS2.1 presented more elongated fruits, with an average fruit shape index of 1.10 which represents an increase of 15.79% as compared to Ved. *FSQP2.1* was delimited from the beginning of chromosome 2 to position 16.42 Mb (Supplementary Fig. S3). This QTL does colocalize with the locus *a* previously mentioned^52^. However, both Ved and PS are andromonoecious, indicating that the genetic variation causing fruit shape differences may be due to a different underlying gene. PS4.4 carried flatter fruits with a fruit shape index of 0.87, a reduction of 11.22% as compared to Ved (Table 1 and Fig. 5). *FSQP4.4* was located on the distal part of chromosome 4, from 27.25 to 34.32 Mb (Supplementary Fig. S3), colocalizing with a recently reported QTL for fruit shape in an F_2_ melon population^53^.

Curiously, none of the ILs fruit weight QTLs were identified in the Ved × PS RIL population^30^. The discrepancy may be due to QTL × QTL interactions, which could make some QTLs indetectable in certain genetic backgrounds. Similarly, fruit weight QTLs detected in the RIL population where not detected in the ILs, but *FWQU5.1* and *FWQU8.1,* would correspond to ILs PS5.2 and PS8.1; PS5.2 presented a high variation between replicates, possibly preventing from detecting a QTL at significant levels. PS8.1, already mentioned, showed an increase in fruit weight of ~ 180 g, considerably higher to the 92.4 g additive effect of PS observed in the Ved × PS RIL population^30^. The high variation between replicates and its limited number could cause lack of statistical power to detect a QTL confidently, but the observed trend in fruit weight suggests that the PS allele in PS8.1 contributes to larger fruits, in agreement with the Ved × PS RIL population. In addition, a QTL for fruit weight was also detected in the same region of chromosome 8 in an F_2_ obtained by crossing a wild and a cultivated *agrestis* accessions, as well as a domestication sweep in a diversity panel of accessions^41^. Additional QTLs on chromosomes 6, 7 and 11 detected in the RIL population, where the Ved allele reduced fruit size, were undetectable in our IL collection. However, the fruit shape QTL on chromosome 2 was identified in both populations.

#### Fruit ripening

Melon has emerged as an alternative model to decipher the genetic control of fruit ripening, since both climacteric and non-climacteric varieties are found within the species. PS is a non-climacteric variety and Ved is highly climacteric and produces a large amount of ethylene at the onset of ripening^54,55^. The hypothesis of a quantitative composition of climacteric ripening, with the ethylene production and associated phenotypes varying in a continuum spectrum, rather than a qualitative presence-absence, is gaining relevance in recent studies^9,31^. The PS IL collection added evidence to this hypothesis, since all ILs from the collection were climacteric. Nearly all the harvested fruits were aromatic and produced abscission layer, two of the most evident and typical traits of climacteric melons. However, PS7.1 did not present consistently the change of color due to chlorophyll degradation during ripening. As mentioned before, a major gene controlling rind color in immature fruit is located on the proximal arm of chromosome 7. Both PS7.1 and PS7.2 carried the PS allele of *Wi*, conferring green color during fruit development. We observed that PS7.2 degraded chlorophyll and turned yellow; this phenotype is completely different from Ved, whose white color turns to cream/light orange during ripening, and from PS, whose green color is maintained during ripening. Interestingly, not all PS7.1 fruits turned to yellow but some stayed green during ripening (Supplementary Fig. S4). We defined the gene *CDQP7.1* for chlorophyll degradation on chromosome 7, from the beginning of the chromosome to 2.62 Mb.

In addition to the presence-absence of climacteric symptoms, we also recorded its earliness (ECD, EARO, EALF), earliness of harvest (HAR) and flesh firmness (FIR) after harvest to quantify the intensity of climacteric ripening.

**Figure 6:**
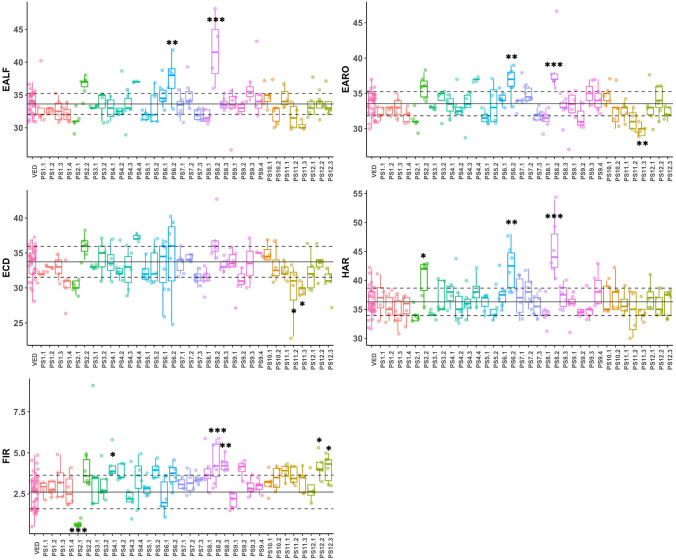
Boxplot representing the values of climacteric ripening traits for ILs. Each dot represents a replicate, the black solid line corresponds to the average and the grey dashed lines to the average ± SD of the recurrent parent Ved. Significant differences are marked with asterisks: *< 0.05, **< 0.01, ***< 0.001.

PS8.2 showed the most delayed and the less intense climacteric characteristics in the IL collection. It presented a delay of 3–8 days in the appearance of climacteric symptoms, from ~ 33 DAP in Ved to ~ 39 DAP in PS8.2, representing about a 20% reduction. The increase in flesh firmness was as high as 48.5%. The QTL was defined from 6.89 to 25.00 Mb on chromosome 8 (Fig. 6, Table 1 and Supplementary Fig. S3).

PS6.2 also presented a delayed ripening of about 4 days, although the earliness of chlorophyll degradation and flesh firmness were not affected. The QTL was delimited from 7.47 to 38.30 Mb on chromosome 6 (Fig. 6, Table 1 and Supplementary Fig. S3).

PS11.2 showed differences in ECD and PS11.3 showed differences in EARO and ECD (Fig. 6 and Table 1). Interestingly, the PS introgressions advanced the appearance of aroma and chlorophyll degradation by ~ 3 days, being the only QTL for which the PS allele enhances a climacteric characteristic, although the effect was subtle.

PS2.2 presented a slight yet significant delay in harvest date, representing ~ 11% increase in comparison to Ved (Fig. 6 and Table 1). A QTL affecting earliness of ethylene production and fruit abscission was identified in the Ved × PS RIL population in a similar region of chromosome 2^31^.

Six lines, PS2.1, PS4.1, PS8.2, PS8.3, PS12.2 and PS12.3, differed from Ved on flesh firmness, although their climacteric behavior was otherwise unaffected. PS2.1 showed a huge decrease in flesh firmness (Fig. 6, Table 1). Cell wall degradation and the subsequent decrease of flesh firmness is only partially dependent on ethylene in melon^34^ and flesh firmness in Ved and PS is similar (Supplementary Fig. S1A). So, this QTL could be the main factor controlling cell wall degradation and allowing the softening of the flesh in the non-climacteric PS. *FIRQP2.1* is located at the beginning of chromosome 2 (0–16.42 Mb) (Supplementary Fig. S3). The other five lines presented an increase in flesh firmness of around ~ 55–70% when compared to Ved, suggesting a complex inheritance of this trait. PS8.3 presented an increase in flesh firmness of about 65%, similarly to PS8.2, with which shares ~ 7 Mb. However, the overall reduction of climacteric intensity in PS8.2 was not observed in PS8.3. We cannot discern whether the *FIRQP8.3* QTL causing this reduction in flesh firmness is exclusive of PS8.3 or shared between both lines, so the overlapping region was included in the interval of the QTL, namely from 25.0 to 34.77 Mb (Supplementary Fig. S3). PS12.2 and PS12.3 also showed an increase in flesh firmness, being *FIRQP12.2* located at the end of chromosome 12 (22.98–25.89 Mb). In this region, two QTLs for flesh firmness were previously described using both genome-wide association study (GWAS) and biparental mapping in melon^56^.

Several QTLs related to climacteric ripening have been already mapped in melon^20,23,55^. In the Ved × PS RIL population, several QTLs controlling or modulating ethylene production and/or climacteric traits as aroma production, fruit abscission and chlorophyll degradation have been identified^31,32^. In contrast with the low rate of overlapping observed for fruit morphology QTLs, all mapped QTLs affecting climacteric ripening were identified using both the Ved × PS RIL population and the PS IL collection, in similar genomic intervals^31^. Among them, *ETHQV8.1*, carried by PS8.2, was clearly and consistently the most relevant in both populations, presenting a delay in both the ethylene peak (measured only in the RIL population) and the appearance of climacteric traits (quantified in the RIL and IL collections). PS6.2 also showed a consistent delay in the climacteric related traits. A gene on chromosome 6 already cloned, *MELO3C016540* (*CmNAC‐NOR*), could be the responsible for the PS6.2 phenotype, since it delays fruit ripening and ethylene biosynthesis when mutated in a climacteric genetic background, and triggers a climacteric response in PS when introgressing the allele of the low climacteric variety PI161375^9^.

Other melon IL collections have explored the genetic basis of fruit ripening^20,21,23^. In total, they mapped two more ripening QTLs on chromosomes 3, and 10. These populations shared one parent with our IL collection, and yet we were able to identify additional QTLs on chromosomes 7 (*CDQP7.1* for chlorophyll degradation, Supplementary Fig. S4), 8 and 11 (several ripening traits, Table 1, Supplementary Fig. S3), supporting the complex genetic architecture of fruit ripening in melon. In addition, the partial effect of some of these QTLs, as observed in PS7.1, may be useful in breeding programs, allowing modifying only the desired trait without altering overall ripening behavior.

## Conclusion

We have developed a new IL collection, using as parental lines two elite European cultivars. A RIL population derived from the same parental lines has proven its effectiveness to identify relevant fruit quality QTLs. Multiple QTLs and major genes controlling several aspects of fruit quality have been identified and the germplasm generated in this work could ease further fine mapping and positional cloning. In addition, these lines could be used to pyramid QTLs controlling complex traits such as fruit size or climacteric ripening, allowing to better understand the genetic interactions among them. Lastly, the PS IL collection may be an excellent resource for breeding programs. Generally, ILs are developed using wild or exotic material, which carry detrimental alleles by linkage drag. By using two commercial varieties of high quality and yet segregating for many relevant traits, we avoided the unfavorable alleles that could be linked to the target gene in other non-commercial materials.

## Materials and methods

### Plant material and breeding scheme

Seedlings were planted in fertilpots and maintained under controlled conditions in an indoor greenhouse facility (CRAG, Barcelona) during approximately three weeks. After this period, selected plants were grown in a greenhouse in Caldes de Montbui (Barcelona) during spring and summer seasons (April–October). Plants were pruned weekly and pollinations were executed manually, allowing to develop only one fruit per plant.

The IL collection was founded by a cross between two commercial varieties, “Védrantais” (Ved), a French melon from the *cantalupensis* group used as female, and “Piel de Sapo” T111 (PS), a Spanish melon from the *inodorus* group used as male. These two varieties segregate for many traits such as fruit morphology, fruit quality and ripening behavior (Table 2). Pollen from the F_1_ plants was used to pollinate female flowers from Ved, obtaining BC_1_ seed with the cytoplasm from the recurrent parent. After BC_1_, Ved was generally used as male and the pre-IL as female. Seedlings of the BC_1_ progenies were screened and a subset of plants was selected following these hierarchical criteria: (1) the complete genome of PS should be contained at least twice in the selected individuals; (2) the lines should carry the lowest possible number of introgressions; (3) the lines should contain the highest possible percentage of Ved genome. The chosen individuals were backcrossed again to obtain the BC_2_ progeny. Analogous cycles of screening and selection were performed for the subsequent progenies following the breeding scheme presented in Fig. 1. When the lines contained three or less introgressions, they were self-pollinated to identify lines carrying a unique introgression in homozygosity within the progeny. Depending on the introgression line, three or four backcrosses followed by one or two self-pollinations were needed to obtain the target introgression line in homozygosity without additional non-target introgressions in other chromosomes.

**Table 2:** Phenotypic traits evaluated in the IL collection.

Category	Trait (units^a^)	Code
Fruit quality	Presence of sutures	SUT
	Rind color of immature fruit	ECOL
	Mottled rind	MOT
	Yellowing of the rind	YELL
	Soluble solids content (°Brix)	SSC
	Flesh color	FC
Fruit morphology	Fruit weight (g)	FW
	Fruit area (cm^2^)	FA
	Fruit perimeter (cm)	FP
	Fruit width (cm)	FWI
	Fruit length (cm)	FL
	Fruit shape	FS
Climacteric ripening	Chlorophyll degradation	CD
	Earliness of chlorophyll degradation (DAP)	ECD
	Aroma production	ARO
	Earliness of aroma production (DAP)	EARO
	Earliness of abscission layer formation (DAP)	EALF
	Earliness of harvesting (DAP)	HAR
	Flesh firmness (kg cm^−2^)	FIR

^a^*DAP* days after pollination.

### DNA extraction and genotyping

DNA extractions were performed from young leaves following the CTAB protocol^57^ with some modifications^30^.

The progenies of BC_1_, BC_2_ and most part of the BC_3_ seedlings were analyzed with an initial set of 48 SNPs (set 1) homogeneously distributed within the 12 melon chromosomes (Supplementary Table S5a). The selected plants from BC_2_ and BC_3_ generations were subsequently genotyped with an additional set of 48 SNPs (set 2) (Supplementary Table S5b). SNPs were identified and designed from resequencing data of both parental lines^58^ and their positions are referred to the melon reference genome v3.6.1 (http://www.melonomics.net). SNPs producing sub-optimal genotypes were substituted by other SNPs located nearby. The progenies of BC_3_S_1_, BC_4_, BC_3_S_2_ and BC_4_S_1_, screened in 2017, were genotyped with the complete set of SNPs (Supplementary Table S5c), which was comprised mostly by sets 1 and 2. Additional SNPs were used whenever necessary to genotype overlapping introgressions (Supplementary Table S5d). Plants were genotyped with SNPs using the KASPar SNP Genotyping System (KBiosciences, Herts, UK). KASPar primers were designed following LGC Genomics protocol. The genotyping was performed using the high-throughput genotyping system Biomark HD, based on the Fluidigm technology, with 48 × 48 (2015 and 2016) and 96 × 96 (2017) chips. In the last phases of screening and selection, when most part of the genome was already fixed for Ved, only SNPs contained within the known introgressions were genotyped with the same KASPar primers but using qPCR in a LightCycler 480 Real-time PCR System, according to the technical instructions provided by the supplier (Roche Diagnostics, Spain).

To calculate the size of the introgressions and the intervals delimiting the QTLs, two assumptions were done; we supposed that the haplotypes of the non-genotyped extremes of the chromosome were the same than the first or last SNP genotyped; and we used the intermediate position between two genotyped SNPs as the virtual recombination breakpoint. The approximate genetic size of the introgressions was calculated using as a reference a genetic map obtained from a Ved × PS RIL population^30^.

### Experimental design and phenotyping of fruit quality and climacteric ripening traits

Phenotyping of most part of the IL collection, 29 ILs, was performed in greenhouse conditions in Caldes de Montbui (Barcelona) during the summer of 2018. The experiment consisted of randomized blocks including from five to seven plants of each IL and 13 plants of the recurrent parent, Ved. Three ILs that were not fully developed in 2018 were grown and phenotyped under the same conditions, along with the Ved controls, in the summer of 2019. Lastly, two more ILs were obtained in 2019 and phenotyped in 2020.

Qualitative traits related with fruit appearance were visually inspected and recorded as presence or absence (Table 2). These traits were rind color of immature fruit (ECOL), yellowing of the rind (YELL), presence of sutures (SUT) and mottled rind (MOT). Flesh color of mature fruits was recorded as orange, white or green. All fruits were weighed (FW), photographed and scanned at harvest. The scanned images were analyzed in Tomato Analyzer 3.0^47^ to extract the values of morphological traits: fruit perimeter (FP), fruit area (FA), fruit length (FL) and fruit width (FWI); fruit shape (FS) was calculated as FL/FWI. In addition to visual inspections, flesh color was also measured in scanned images in Tomato Analyzer. The CIELAB color parameters were used to estimate an index, FC, that represents the differences of each IL with respect to Ved. FC was calculated as the summatory of the subtraction of the average a, b and L values of the IL minus the average a, b and L values of Ved, respectively. To measure the soluble solid content (SSC), four 1-cm cylinders of flesh were obtained from the central part of the fruit in a proximal–distal section and the SSC value was estimated by a hand refractometer from manually extracted juice.

Ripening-related traits were evaluated as qualitative (presence or absence) or quantitative (earliness of appearance of the trait in days after pollination, DAP). The visual inspection of melon fruits attached to the plant was performed daily, from approximately 25 DAP until harvest. Chlorophyll degradation (CD) and its earliness (ECD) were recorded when degreening was obvious in visual inspections. Aroma production (ARO) and its earliness (EARO) were evaluated every other day by smelling the fruits. Earliness of abscission layer formation (EALF) was recorded as well after daily visual inspection when a scar in the pedicel was clearly formed. The harvest date was determined by the following criteria and registered (HAR): (a) abscission date when the fruit abscised; (b) after five days of the appearance of the abscission layer; (c) at 55–60 days after pollination when fruits were non-climacteric. The firmness of fruit flesh (FIR) was measured at harvest using a penetrometer (Fruit Test™, Wagner Instruments), in at least three regions of the fruit (distal, proximal and median), and the mean value was registered.

### QTL and major gene mapping and statistical analyses

A QTL was designated when the mean of one or more ILs carrying the corresponding region was significantly different to Ved mean using Dunnett’s test. When two ILs overlapped in a certain region of the introgression, the overlapping interval defined the QTL if both lines were significantly different to Ved but was excluded when only one line presented the phenotype. The statistical analysis was implemented in R (version 3.6.3)^59^ using the functions aov and glht from the packages stats and multcomp, respectively. Three levels of significance (0.05, 0.01 and 0.001) were used and the p-values were specified. The genomic intervals of the QTLs and major genes were calculated using the intermediate position between two genotyped SNPs as the virtual recombination breakpoint.

Most ILs were evaluated in 2018, however three ILs were evaluated in 2019 and two more in 2020. To add these lines to the analyses, we first tested whether the recurrent parent Ved was affected by the environment. For most traits, no significant differences were observed between years. Only size-related traits (FW, FA, FP, FL and FWI) were affected. Consequently, for the morphological traits, a separated analysis was performed for each year (Fig. 5 and Supplementary Figs. S2 and S5). For SSC, FC and ripening traits (Figs. 4 and 6), the data from 2018, 2019 and 2020 were merged.

As the assumptions for applying the Dunnett test (normality of the data and equal variances) were not always accomplished (Supplementary Table S6a), we re-analyzed the data using the non-parametric Mann–Whitney test (Supplementary Table S6b). As 74% of the QTLs detected with the Dunnett test were significant with the non-parametric test, we presented the QTLs detected with Dunnett test in Fig. 1.

### Plant material

The collection and use of plant material complies with institutional, national, and international guidelines and legislation. Védrantais and Piel de Sapo T111 are melon inbred lines gently provided by Semillas Fitó SA.

## Supplementary Information

Supplementary Information 1.

Supplementary Information 2.

Supplementary Information 3.

Supplementary Information 4.

Supplementary Information 5.

## Data Availability

Relevant data are submitted as “Supplementary material” or are available upon request.
