# Supplementary material for: A novel introgression line collection to unravel the genetics of climacteric ripening and fruit quality in melon
Source: Sci Rep. 2021 May 31;11:11364. doi: 10.1038/s41598-021-90783-6 (PMC8166866; doi:10.1038/s41598-021-90783-6)
Supplement: Supplementary file 1 — Supplementary Information 1. [file 41598_2021_90783_MOESM1_ESM.pdf]

A novel introgression line collection to unravel the genetics of climacteric ripening and fruit quality in melon

Lara Pereira[1$], Miguel Santo Domingo[1$], Jason Argyris[1,2], Carlos Mayobre[1], Laura Valverde[1], Ana Montserrat Martín-Hernández[1,2], Marta Pujol[1,2*], Jordi Garcia-Mas[1,2*]

Supplementary information

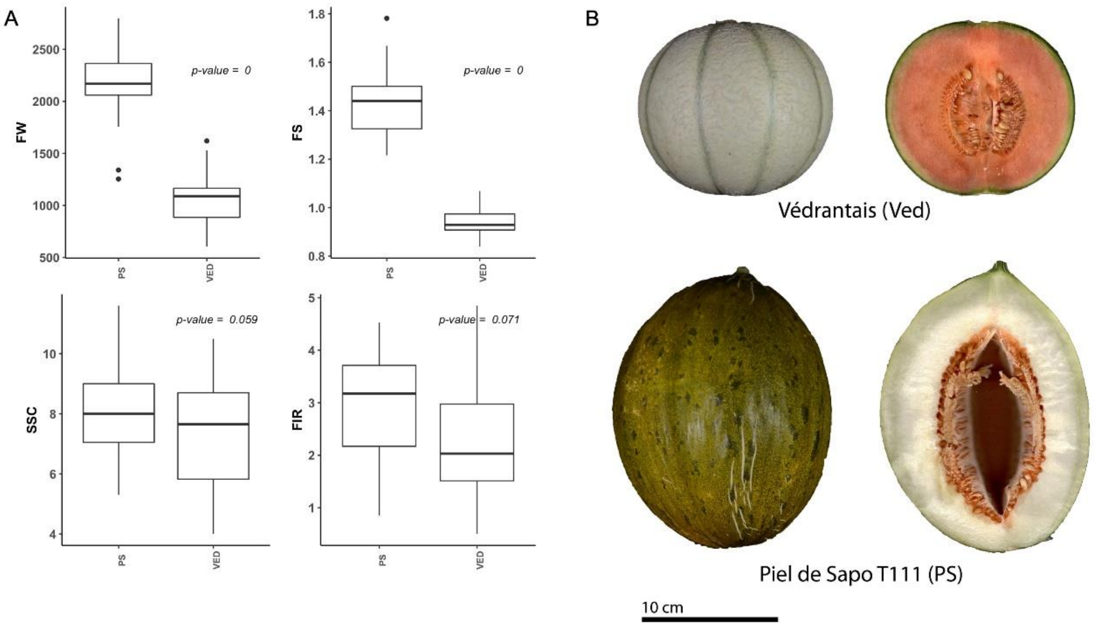

Figure S1 A. Phenotypic differences (FW, FS, SSC and FIR) between the parental lines used to develop the IL collection from the evaluation performed in 2020 B. Images of the parental lines used to develop the IL collection.

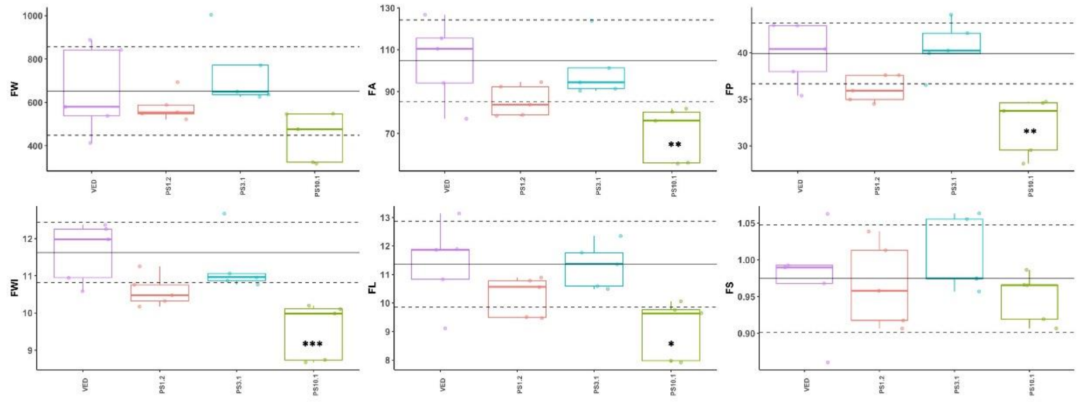

Figure S2 Boxplot representing the values of morphological traits (FW, FA, FP, FWI, FL and FS) for ILs evaluated in 2019. Each dot represents a replicate, the black solid line corresponds to the average and the grey dashed lines to the average ± SD of the recurrent parent Ved. Significant differences are marked with asterisks: * <0.05, ** <0.01, *** <0.001.

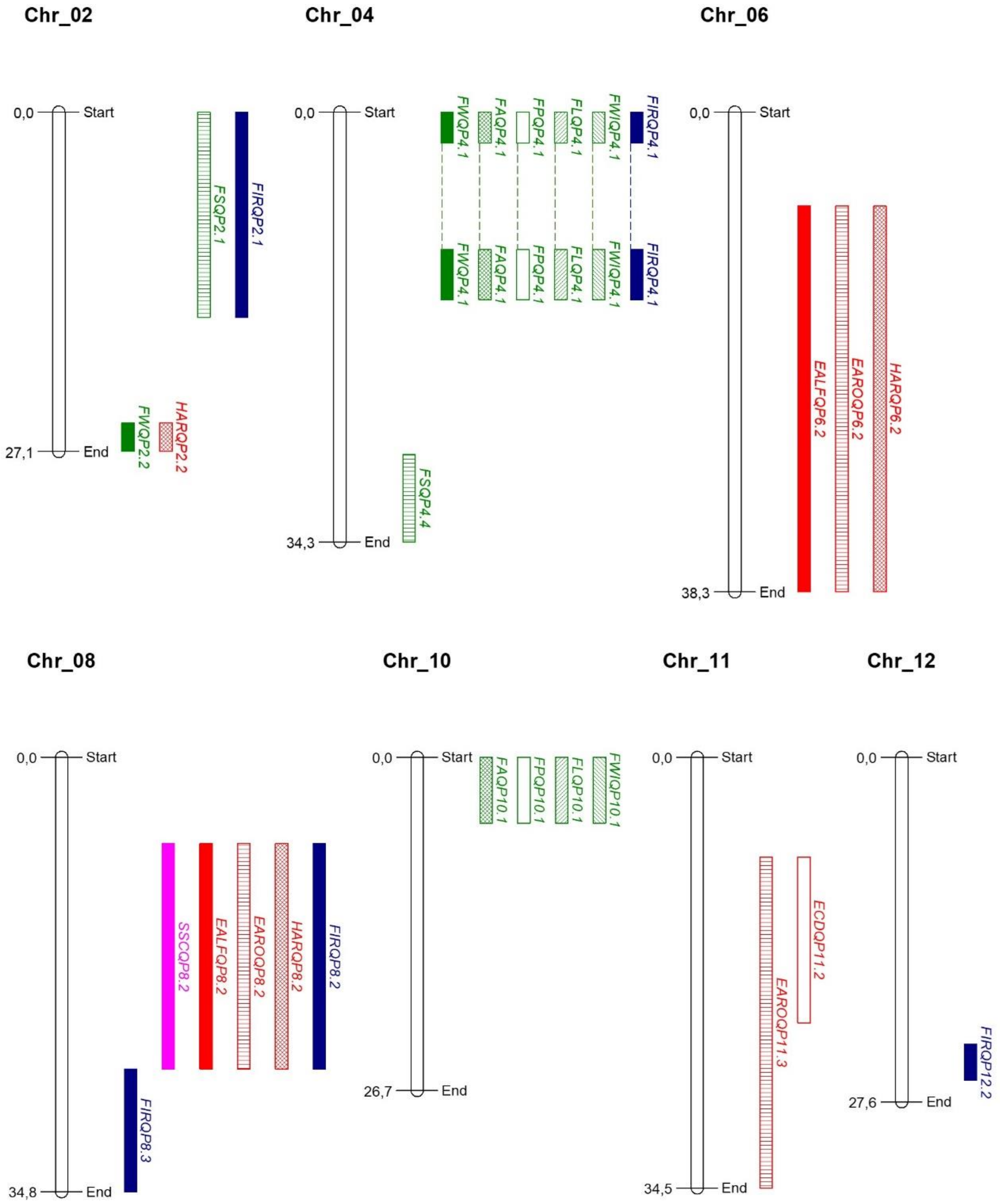

Figure S3 Physical map containing detected QTLs. QTLs are represented as colored bars using the genomic interval in Mb. Green tones and patterns for morphological QTLs (FW, FA, FP, FL FWI and FS), red tones and patterns for climacteric traits (EALF, EARO, ECD and HAR), dark blue for FIR and purple for SSC. The two putative positions of *FWQP4.1* are linked by a dashed line.

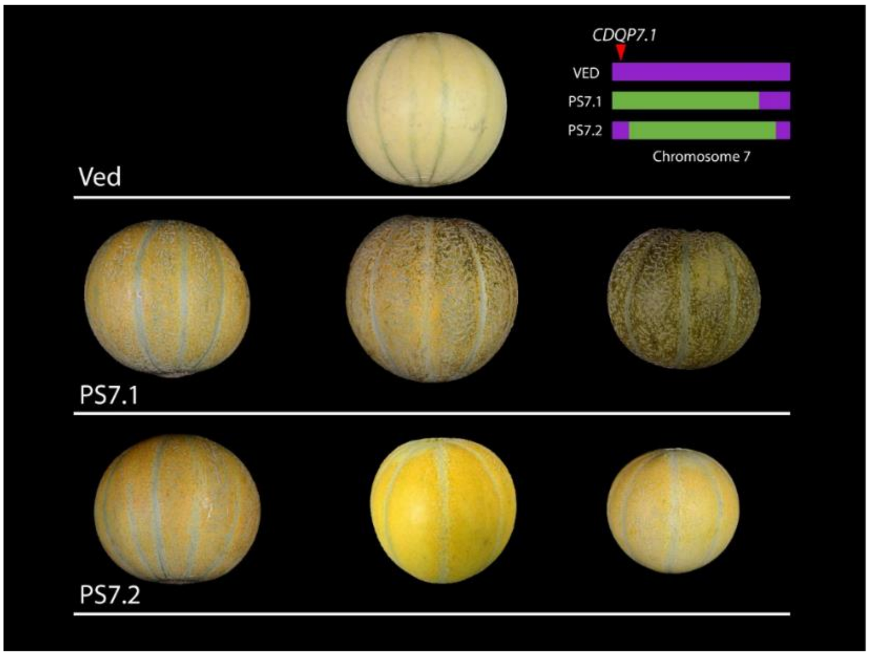

Figure S4 Effect of the QTL *CDQP7.1* on chlorophyll degradation.

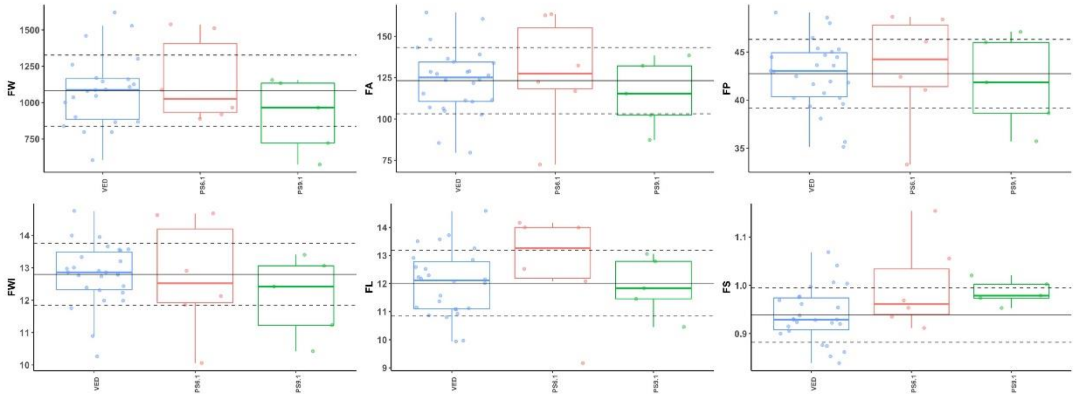

Figure S5 Boxplot representing the values of morphological traits (FW, FA, FP, FWI, FL and FS) for ILs evaluated in 2020. Each dot represents a replicate, the black solid line corresponds to the average and the grey dashed lines to the average ± SD of the recurrent parent Ved. Significant differences are marked with asterisks: * <0.05, ** <0.01, *** <0.001

Table S1 Genotypes of the IL collection

See additional .xls file

Table S2 Description of the IL collection

Chr	Number of ILs	Introgression size (Mb)			Introgression size (cM)		
		Average	Min	Max	Average	Min	Max
1	4	15,43	2,46	32,02	53,4	22,1	82,2
2	2	13,53	2,29	24,77	63,9	14,7	113,0
3	2	22,69	21,76	23,62	77,1	75,8	78,4
4	4	15,00	8,53	19,40	68,5	44,2	86,6
5	2	14,66	2,76	26,57	62,6	29,3	95,8
6	2	33,07	30,83	35,32	109,8	90,5	129,2
7	3	15,69	2,98	23,36	56,9	30,2	77,2
8	3	14,21	6,89	25,98	51,2	37,2	58,8
9	4	18,24	14,91	20,52	46,0	17,6	70,8
10	2	19,22	17,98	20,47	59,7	24,9	94,6
11	3	24,94	18,56	29,79	71,5	29,7	105,3
12	3	17,11	4,58	23,75	59,1	47,1	73,5
**Total**	**34**	**18,14**	**2,29**	**35,32**	**62,8**	**14,7**	**129,2**

Table S3 General description of some IL collections

Species	Donor parental	Needed generations[1]	ILs/ chr[2]	First MAS-generation	Population used in the genetic map	Reference
Tomato	*S. pimpinellifolium*	6	4.6	BC2	BC2	(Barrantes et al. 2014)
Eggplant	*S. incanum*	7	2.1	BC1	BC1	(Gramazio et al. 2017)
Peach	Almond "Texas"	3	3.5[2]	BC1	F2	(Serra et al. 2016)
Strawberry	*Fragaria bucharica*	4	6	BC1	F2	(Urrutia et al. 2015)
Melon	PI 162375 "Songwhan Charmi"	6	4.75	BC1	F2, DHL	(Eduardo et al. 2005)
Melon	PI 420176 "Ginsen Makuwa"	6	1.3	BC2	F2, DHL	(Perpiñá et al. 2016)
Melon	PI 273438 "Queen's pocket melon"	6	1.3	BC2	DHL	(Castro et al. 2019)
Melon	"Piel de Sapo" T111	6	2.8	BC1	RIL	This work

Table S4 Average and SD values of phenotypic traits analyzed in the IL collection and the parental lines PS and Ved, and raw phenotypic data.

See additional .xls file

Table S5 Information about the SNPs used to develop and genotype the IL collection. A. Set 1 of SNPs. B Set 2 of SNPs. C Complete set of SNPs. D Additional SNPs.

See additional .xls file

Table S6 Tests applied to check the assumptions for the Dunnet test. A. p-values obtained in the Shapiro test to check normality of the data. If p-value < 0.05 (bold numbers), it is not considered normal. Some data cannot be tested because the lack of variability in the replicates. B. QTLs detected using the non-parametric Mann-Whitney test of each IL against Ved, compared with the QTLs detected with Dunnnet's test (shown in Table 1). p-values were adjusted using the Holm-Bonferroni method.

See additional .xls file
